# Thalidomide for the Management of Bleeding Episodes in Patients with Hereditary Hemorrhagic Telangiectasia: Effects on Epistaxis Severity Score and Quality of Life

**DOI:** 10.4274/tjh.galenos.2018.2018.0190

**Published:** 2019-02-07

**Authors:** Mehmet Baysal, Elif G. Ümit, Hakkı Onur Kırkızlar, Ali Caner Özdöver, Ahmet Muzaffer Demir

**Affiliations:** 1 Trakya University Faculty of Medicine, Department of Hematology, Edirne, Turkey

**Keywords:** Hereditary hemorrhagic telangiectasia, Thalidomide, Epistaxis, Quality of life

## Abstract

Hereditary hemorrhagic telangiectasia (HHT) is a rare autosomal dominantly inherited disorder characterized by bleeding episodes. These episodes tend to happen spontaneously and reduce the quality of life. Patients are often unresponsive to local measures. With the pathophysiological role of angiogenesis in HHT, antiangiogenic drugs including thalidomide are used to control bleeding episodes. In our study, we evaluated 6 patients with HHT, calculating their Epistaxis Severity Score (ESS) and performing a quality of life assessment with the 36-Item Short Form Health Survey Questionnaire (SF-36), and we studied the alterations of these evaluations with thalidomide treatment. Three patients were male and three were female. Mean age was 60.50 years. No side effects were observed during the treatment period. Improvements of certain SF-36 dimensions including physical functioning, physical component summary, and mental component summary and of the ESS were observed after treatment. Thalidomide may be effective to control bleeding episodes with a reasonable tolerance profile in patients with HHT.

## Abstract (tr)

Herediter hemorajik telenjiektazi (HHT), kanama atakları ile karakterize, nadir görülen, otozomal dominant kalıtılan bir hastalıktır. Bu ataklar spontan olarak geçekleşmekte ve hastların yaşam kalitesini düşürmektedir. Hastalar genellikle lokal cerrahi müdahalelere yanıtsızdır. HHT’deki anjiyogenezin patofizyolojik rolü ile kanama ataklarını kontrol etmek için talidomid içeren antianjiyojenik ilaçlar kullanılması düşünlmüştür. Çalışmamızda 6 HTT’li hasta değerlendirilmiştir. Hastaların talidomid tedavisi öncesinde ve sonrasında Epistaksis Ciddiyet Skoru (ESS) ve yaşam kalitesini değerlendirmek amacıyla 36 maddelik Kısa Sağlık Durumu Anketi (SF-36) değerlendirildi. Hastalarımızın 3’ü erkek olup, 3’ü kadındı. Ortalama yaşları 60,50 idi. Tedavi süresince ciddi bir yan etki izlenmedi. Tedaviden sonra ESS ve SF-36’nın fiziksel fonksiyon, fiziki bileşen özeti, ruhsal bileşen özeti gibi alt gruplarında önemli düzeyde iyileşmeler izlendi. Talidomide HHT hastalarında kanama atakların kontrol etmede makül bir yan etki profiliyle etkili olabilir.

## Introduction

Hereditary hemorrhagic telangiectasia (HHT) is a rare autosomal dominant disorder characterized by telangiectasia and arteriovenous malformations of the skin, mucosal tissues, and internal organs including the gastrointestinal tract, liver, and lungs. Its prevalence is estimated to be between 1/5000 and 1/10,000 worldwide. Recurrent epistaxis due to nasal telangiectasia is the most common finding [1,2]. There are several mutations linked with the disease and the most frequent mutations are reported as the *ENG* gene encoding endoglin and the *ACVRL1* gene encoding activin A receptor type II-like kinase 1 [3]. Anemia is a very common symptom in HHT patients, not only due to bleeding from telangiectases located in the nasal mucosa but also to telangiectases located in the gastrointestinal tract, especially active in older ages. The diagnostic criteria for HHT were defined in 2000 and updated in 2011, including epistaxis, telangiectases, vascular malformations, and family history. The presence of 3 of these criteria is suggested to be sufficient for diagnosis [4]. Since epistaxis is the most common manifestation, patients are frequently admitted and evaluated by otolaryngologists. Invasive measures and interventions including cauterization are ineffective and the diagnosis of HHT, which is quite rare, comes into consideration. Before the identification of pathogenetic mechanisms of bleeding from telangiectases, local or systemically effective antifibrinolytics (tranexamic acid, epsilon aminocaproic acid), estrogen or progesterone preparations such as ethinylestradiol or norethisterone, and selective estrogen receptor modulators such as mestranol, norethynodrel, tamoxifen, and raloxifene have been used to control recurrent epistaxis and gastrointestinal bleeding episodes [5]. Thalidomide was first designed as a sedative and then used for nausea in pregnant women; it has been responsible for about 10,000 infants being born with phocomelia. This disastrous experience led to the further understanding of the mechanism of action of thalidomide. The effects of thalidomide on the innate and adaptive immune system as well as tumor development and angiogenesis have made thalidomide a model drug for the development of anticancer treatments. Besides the role of antiangiogenic medications in tumor genesis and cancer treatment, thalidomide and other novel endothelial and vascular growth factor inhibitors including bevacizumab are suggested to be beneficial in patients with HHT since the pathogenesis of HHT primarily relies on excessive formation of new vessels [6,7,8,9,10,11,12,13]. This has been supported with limited evidence, however, due to the rare incidence of HHT itself. In our study, we aimed to evaluate our thalidomide experience based on a bleeding score developed in a multicenter study with a large cohort of HHT patients [14] as well as their improvement in quality of life as assessed with a valid and internationally accepted scale, the 36-Item Short Form Health Survey Questionnaire (SF-36) [15].

## Materials and Methods

The data of six patients diagnosed with HHT according to the Curaçao HHT diagnostic criteria were recorded from their files in a retrospective manner. All patients were admitted and treated by otolaryngologists with simple epistaxis first, with cauterization and local measures like tampons and compression, and as they were observed to be refractory to such interventions they were referred to our department with consideration of a possible HHT diagnosis. All patients were refractory to local cauterization and local or systemically effective antifibrinolytics. All patients were anemic due to chronic bleeding episodes. Heavy bleedings due to nasal telangiectases were assessed with the Epistaxis Severity Score (ESS) [15], while the requirement of transfusions was based on the 2016 American Association of Blood Bank clinical guidelines on red blood cell transfusion [16]. Treatment of bleeding episodes with thalidomide was started at 50 mg daily and dose escalation to 100 mg/daily was scheduled individually according to response. Patients were evaluated every 2 months for response assessment. Therefore, all patients started with 50 mg/daily and Patient 1, Patient 2, Patient 3, and Patient 4 were escalated to 100 mg/daily after 2 months. Patient 5 and Patient 6 did not need to escalate to 100 mg and continued with 50 mg/daily. Treatment duration was based on controlling epistaxis episodes, reducing the ESS, and improving anemia. Quality of life of the patients was assessed with the SF-36, the most commonly used such survey, which consists of two main dimensions: a physical component summary, including physical function, physical role, pain, and general health status, and a mental component summary, including social functioning, emotional role, and mental health. The scale is a self-assessment scale and is filled in by the patient in a very short time [15]. This study was approved by the Trakya University Medical Faculty’s Ethics Committee of Non-Invasive Clinical Research (2017-334). Written informed consent was obtained from all patients. Statistical calculations were performed using SPSS 22 for PC (IBM Corp., USA). The Wilcoxon signed ranks test was used for nonparametric data for two related samples.

## Results

**Figure 1:**
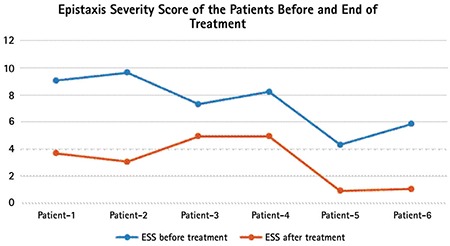
Epistaxis Severity Score of the patients before and at the end of treatment.

Three patients were male and three were female. Mean age was 60.50±10.07 years (range: 44-74). The mean ESS score before treatment was 7.40±2.02 (range: 4.31-9.66) and after treatment it was 3.10±1.79 (range: 0.92-4.94). This finding was statistically significant (p=0.028). The change of hemoglobin level (g/dL) was also statistically significant before and after treatment (p=0.027) (Table 1). One patient reported grade one dizziness and one patient reported nausea, which resolved spontaneously without a need for drug withdrawal. Patients were frequently asked about and examined for common side effects of thalidomide such as neuropathy and constipation. No patient reported these side effects, and no patient had neutropenia or hematologic toxicity. Each patient tolerated the drug well. All SF-36 item scores were found to be increased after the treatment, which may be regarded as improvement of quality of life with thalidomide treatment. Improvements of certain SF-36 dimensions including physical functioning, physical component summary, and mental component summary were statistically significant (p=0.042, 0.048, and 0.046 respectively). Detailed assessment of each patient is summarized in Table 2 and Table 3. The change in ESS before and at the end of treatment is demonstrated in Figure 1.

## Discussion

As a rare disease, the prevalence of HHT is much higher than that of inherited bleeding disorders hemophilia A and B with its autosomal dominant nature. As the most common symptoms are associated with bleeding, patients are frequently evaluated by emergency departments or surgical and general practice departments and are regarded as cases of simple nose bleeds or gastrointestinal hemorrhage although an autosomal dominantly inherited disorder is present with a prominent family history that is ignored and unappreciated. The development of telangiectasia is related to aging, and as life expectancy is not affected and is even reported to be increased in recent studies, the quality of life of these patients should be a main goal of treatment [17,18]. Besides the irritative nature of mucosal bleeding, the consequence of these chronic bleeding episodes is iron deficiency and iron-deficiency anemia. Severe recurrent bleeding episodes may even lead to transfusion requirements. As the international guidelines support a restrictive transfusion approach, in our study as well as our daily practice we follow this approach and limit our transfusion practice as transfusion is not indicated until the hemoglobin level is 7-8 g/dL if the patient is hemodynamically stable and asymptomatic. We instead support the patients with iron supplements. In patients with recurrent severe bleeding episodes, parenteral agents such as iron carboxymaltose may be reasonable to also protect the gastrointestinal tract and avoid the irritation of oral iron as well as to limit transfusion. To control bleeding episodes once and for all, the pathogenesis of HHT has been researched. Excessive angiogenesis has been the underlying mechanism of telangiectases and vascular malformations and the inhibition of angiogenesis was sought. This idea of anti-angiogenesis has brought back one of the most unfortunate medical hazards in history: thalidomide for morning sickness in pregnancy, causing phocomelia and other malformations. Several case series and phase two clinical trials with thalidomide reported long-term improvement in epistaxis and also hematological parameters [6,7,8,9,10]. In our study, though with a limited number of patients, we observed beneficial effects of thalidomide to control bleeding episodes, reduce the need to support patients with transfusions, and therefore increase the life quality of our patients. In a recent study, a systematic analysis of 4 studies involving 43 patients with thalidomide reported beneficial effects of thalidomide to control bleeding episodes, though with a comment on the lack of an optimal treatment modality in HHT [6].

## Conclusion

As an underappreciated inherited disease, HHT should be within the top possibilities in the differential diagnosis of patients with recurrent/refractory and debilitating mucosal bleeding. Family history should not be overlooked as it is one solid item of the diagnostic criteria. Besides short-lived supportive measures, patients should be referred to experienced centers for therapeutic modalities.

## Figures and Tables

**Table 1:**
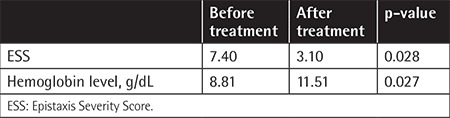
Improvement in Epistaxis Severity Score and hemoglobin levels before and after treatment.

**Table 2:**
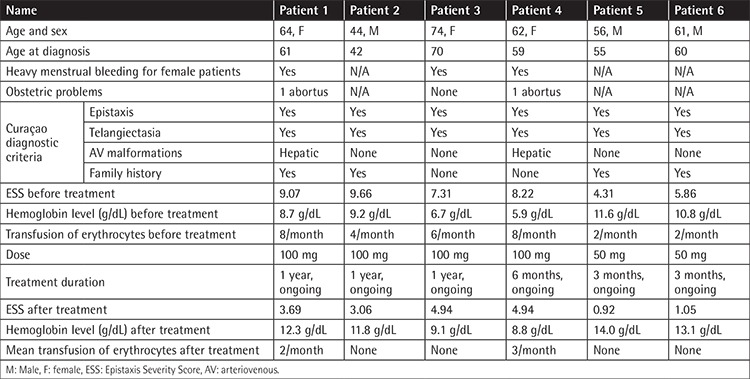
Characteristics of patients and response to thalidomide.

**Table 3:**
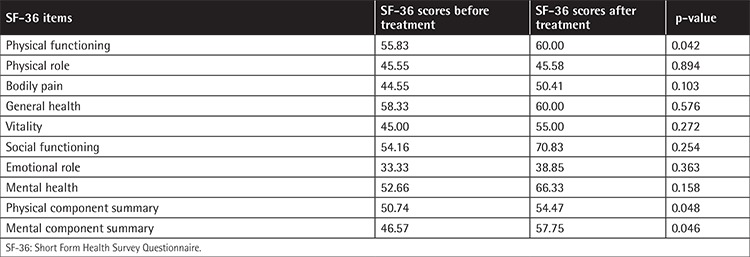
Quality of life assessment and improvement after thalidomide.

## References

[1] Kroon S, Snijder RJ, Faughan ME, Mager HJ (2018). Systematic screening in hereditary hemorrhagic telangiectasia: a review. Curr Opin Pulm Med.

[2] Faughnan ME, Palda VA, Garcia-Tsao G, Geisthoff UW, McDonald J, Proctor DD, Spears J, Brown DH, Buscarini E, Chesnutt MS, Cottin V, Ganguly A, Gossage JR, Guttmacher AE, Hyland RH, Kennedy SJ, Korzenik J, Mager JJ, Ozanne AP, Piccirillo JF, Picus D, Plauchu H, Porteous ME, Pyeritz RE, Ross DA, Sabba C, Swanson K, Terry P, Wallace MC, Westermann CJ, White RI, Young LH, Zarrabeitia R;, HHT Foundation International - Guidelines Working Group (2011). International guidelines for the diagnosis and management of hereditary haemorrhagic telangiectasia. J Med Genet.

[3] Sugden WW, Siekmann AF (2018). Endothelial cell biology of Endoglin in hereditary hemorrhagic telangiectasia. Curr Opin Hematol.

[4] Shovlin CL, Guttmacher AE, Buscarini E, Faughnan ME, Hyland RH, Westermann CJ, Kjeldsen AD, Plauchu H (2000). Diagnostic criteria for hereditary hemorrhagic telangiectasia (Rendu-Osler-Weber syndrome). Am J Med Genet.

[5] Geisthoff UW, Nguyen HL, Röth A, Seyfert U (2015). How to manage patients with hereditary haemorrhagic telangiectasia. Br J Haematol.

[6] Halderman AA, Ryan MW, Clark C, Sindwani R, Reh DD, Poetker DM, Invernizzi R, Marple BF (2018). Medical treatment of epistaxis in hereditary hemorrhagic telangiectasia: an evidence-based review. Int Forum Allergy Rhinol.

[7] Xu M, Hou Y, Sheng L, Peng J (2013). Therapeutic effects of thalidomide in hematologic disorders: a review. Front Med.

[8] Fang J, Chen X, Zhu B, Ye H, Zhang W, Guan J, Su K (2017). Thalidomide for epistaxis in patients with hereditary hemorrhagic telangiectasia: a preliminary study. Otolaryngol Head Neck Surg.

[9] Hosman A, Westermann CJ, Snijder R, Disch F, Mummery CL, Mager JJ (2015). Follow-up of thalidomide treatment in patients with hereditary haemorrhagic telangiectasia. Rhinology.

[10] Lebrin F, Srun S, Raymond K, Martin S, van den Brink S, Freitas C, Bréant C, Mathivet T, Larrivée B, Thomas JL, Arthur HM, Westermann CJ, Disch F, Mager JJ, Snijder RJ, Eichmann A, Mummery CL (2010). Thalidomide stimulates vessel maturation and reduces epistaxis in individuals with hereditary hemorrhagic telangiectasia. Nat Med.

[11] Invernizzi R, Quaglia F, Klersy C, Pagella F, Ornati F, Chu F, Matti E, Spinozzi G, Plumitallo S, Grignani P, Olivieri C, Bastia R, Bellistri F, Danesino C, Benazzo M, Balduini CL (2015). Efficacy and safety of thalidomide for the treatment of severe recurrent epistaxis in hereditary haemorrhagic telangiectasia: results of a non-randomised, single-centre, phase 2 study. Lancet Haematol.

[12] Steineger J, Osnes T, Heimdal K, Dheyauldeen S (2018). Long-term experience with intranasal bevacizumab therapy. Laryngoscope.

[13] Iyer VN, Apala DR, Pannu BS, Kotecha A, Brinjikji W, Leise MD, Kamath PS, Misra S, Begna KH, Cartin-Ceba R, DuBrock HM, Krowka MJ, O’Brien EK, Pruthi RK, Schroeder DR, Swanson KL (2018). Intravenous bevacizumab for refractory hereditary hemorrhagic telangiectasia-related epistaxis and gastrointestinal bleeding. Mayo Clin Proc.

[14] Hoag JB, Terry P, Mitchell S, Reh D, Merlo CA (2010). An epistaxis severity score for hereditary hemorrhagic telangiectasia. Laryngoscope.

[15] Ware JE Jr, Sherbourne CD (Med Care). The MOS 36-item short-form health survey (SF- 36). I. Conceptual framework and item selection..

[16] Carson JL, Guyatt G, Heddle NM, Grossman BJ, Cohn CS, Fung MK, Gernsheimer T, Holcomb JB, Kaplan LJ, Katz LM, Peterson N, Ramsey G, Rao SV, Roback JD, Shander A, Tobian AA (2016). Clinical practice guidelines from the AABB: Red blood cell transfusion thresholds and storage. JAMA.

[17] de Gussem EM, Edwards CP, Hosman AE, Westermann CJ, Snijder RJ, Faughnan ME, Mager JJ (2016). Life expectancy of parents with hereditary haemorrhagic telangiectasia. Orphanet J Rare Dis.

[18] Geerts L, Fantini-Hauwel C, Brugallé E, Boute O, Frénois F, Defrance L, Manouvrier-Hanu S, Petit F, Antoine P (2017). The subjective experience of patients diagnosed with hereditary hemorrhagic telangiectasia: a qualitative study. J Genet Couns.

